# The Miraculous Diagnostic Role of Indocyanine Green in a Diabetic Foot Ulcer: A Rare Case Report

**DOI:** 10.7759/cureus.55525

**Published:** 2024-03-04

**Authors:** Apurv S Wadgaonkar, Sanjay V Deshpande, Nachiket P Rahate, Prashant V Rahate

**Affiliations:** 1 Orthopedics and Traumatology, Jawaharlal Nehru Medical College, Datta Meghe Institute of Higher Education and Research, Wardha, IND; 2 Surgery, Jawaharlal Nehru Medical College, Datta Meghe Institute of Higher Education and Research, Wardha, IND; 3 Surgery, Seven Star Hospital, Nagpur, IND

**Keywords:** stryker, perfusion, diabetic foot ulcer, nir, icg

## Abstract

Diabetes is a chronic metabolic disorder characterized by elevated levels of glucose in the blood. This causes small nerve polyneuropathy resulting in diabetic foot ulcers. A diabetic foot ulcer is an open sore or wound that develops as a result of chronic diabetes. Indocyanine green angiography (ICGA) near-infrared (NIR) can provide real-time visualization of blood flow within the microvasculature of the underlying organ. Here, we discuss a 63-year-old patient who came with a diabetic foot ulcer over his right great toe. His blood glucose level was 208 mg/dl. He drinks alcohol occasionally and smokes regularly. The tissue perfusion of his right foot was checked using the indocyanine green dye, after which orthopedic surgeons were consulted, and the gangrenous part was amputated.

## Introduction

According to the International Diabetes Federation (IDF), India is considered the "diabetes capital of the world" with over 77 million adults aged 20-79 years living with diabetes. This number is projected to increase to 134 million by 2045 if current trends persist [1]. The Indian government has initiated various programs and policies to address the rising burden of diabetes, including the National Programme for Prevention and Control of Cancer, Diabetes, Cardiovascular Diseases and Stroke (NPCDCS) [2]. Diabetic neuropathy is a type of nerve damage that can occur in people with diabetes. It's a common complication of both type 1 and type 2 diabetes. It includes peripheral, proximal, focal, and autonomic neuropathy. Peripheral neuropathy is the most common type of diabetic neuropathy and usually affects the feet and legs first, followed by the hands and arms [3]. Symptoms include numbness, tingling, burning sensations, muscle weakness, and loss of coordination. Peripheral neuropathy can lead to foot ulcers and infections, which, if left untreated, can result in serious complications such as gangrene and amputation. Diabetic foot ulcer is an open sore or wound that develops on the foot, typically on the bottom of the foot which can lead to serious complications, including infection, cellulitis, osteomyelitis, and gangrene [4]. Severe complications may necessitate amputation of the affected toe, foot, or lower extremity [5]. Indocyanine green angiography (ICGA) near-infrared (NIR) can provide real-time visualization of blood flow within the microvasculature of the foot [6]. In cases where surgical intervention is considered for diabetic foot ulcers, ICGA may be used intraoperatively to assess tissue viability and identify regions with compromised blood supply. NIR fluorescence imaging systems typically use light in the NIR spectrum, which ranges from 700 to 900 nanometers [7]. This information can help surgeons make more informed decisions regarding tissue debridement, revascularization procedures, or flap reconstructions. In this case report, we present a case of 63-year-old noncompliant patient with a history of diabetic foot ulcer whose intraoperative diagnostic modality was aided by ICGA-NIR imaging [8].

## Case presentation

**Figure 1:**
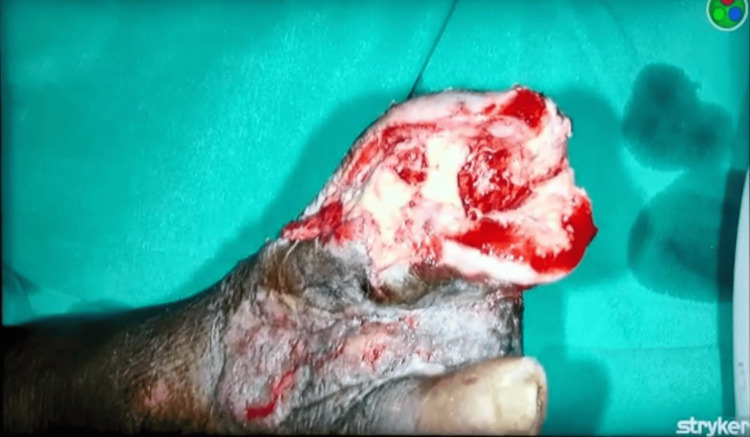
The image depicts diabetic foot ulcer on the right great toe.

A 63-year-old male patient came to the surgery department of a tertiary care hospital in the Vidarbha region of India with complaints of a foul-smelling nonhealing wound on the dorsomedial aspect of the right foot. The patient gave a history of wearing tight uncomfortable footwear which resulted in an unnoticed wound over his right great toe. The wound was not associated with pain, but he came for consultation when he noticed discharge and a foul smell. The patient also had a history of diabetes which was diagnosed 15 years back for which he was prescribed metformin, but he was noncompliant with the same. The patient has no family history of diabetes mellitus. He consumes alcohol socially and smokes cigarettes 12 pack years. On examination, the patient was febrile (38.4°C) and had a blood pressure of 130/80 mm of Hg. On local examination, he had a weak pulse over the anterior and posterior tibial arteries and pitting edema over the medial malleolus of the right ankle. The ulcer was approximately 4 x 3 cm and included his right hallux as seen in Figure 1.

On further investigation, his blood glucose level was 208 mg/dl, and further investigations are enumerated in Table 1.

**Table 1:** Laboratory investigation. WBC count and HbA1C are found to be elevated which is suggestive of diabetes with systemic inflammation. The values are according to source [9]. WBC: white blood cells; HbA1C: glycosylated hemoglobin

Test	Observed value	Reference value
Hemoglobin	10.5 mg/dL	12-15 mg/dL
WBC count	18,500 cells/mm^3^	4,000-11,000 cells/mm^3^
Platelet count	100,000/mm3	150,000-450,000/mm^3^
HbA1C	8.2%	<5.6%

**Figure 2:**
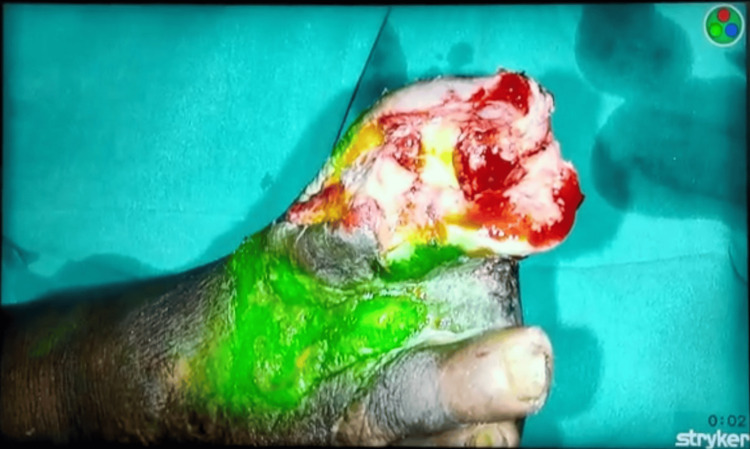
The image shows the green ICG dye taken up by viable tissue. ICG: Indocyanine green

The pre-operative procedure of ICGA-NIR to detect viable and non viable tissue was explained to the patient and consent was acquired for the same. At first a preliminary dose of ICG was administered to the patient to check for any anaphylactic reactions. After the patient was unresponsive to the preliminary dose, he was administered 0.1mg/kg dose of 0.1% ICG dye intravenously approximately 45 minutes before visualizing it under the NIR filter as seen in Figure 2.

**Figure 3:**
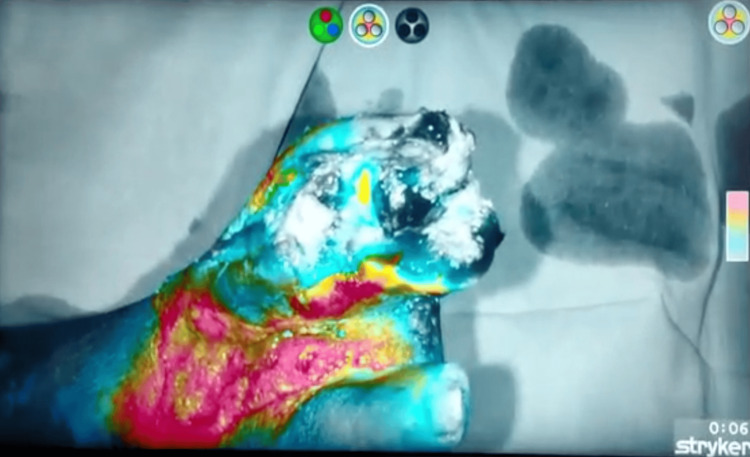
The image shows variable perfusion levels under NIR imaging. NIR: Near-infrared

Stryker Corporation is a globally renowned medical technology company dedicated to enhancing healthcare delivery and patient outcomes which specializes in medical scopes for NIR imaging. ICG dye which was injected can be visualized under NIR light using this Stryker scope. The procedure is interpreted as various color profiles: for example, red color signifies adequate perfusion, blue signifies reduced perfusion, and grey color signifies absence of perfusion. In this patient, it was observed that the tip of the great toe was grey in color and the rest of the toe was blue in color while the distal dorsum of the foot was red in color as seen in Figure 3.

After ICG-NIR imaging, orthopedic surgeons were consulted and transmetatarsal amputation was planned for the patient. The patient was also advised hyperbaric oxygen therapy for the remaining poorly perfused region of the foot.

The patient was called for follow-up four weeks postsurgery. On examination, the surgical site was clean and dry. Physical therapy or rehabilitation was recommended to help restore strength, flexibility, and function to the foot and ankle gradually. Regular follow-up visits were advised to the patient to monitor the progress of healing, assess for any complications, and make adjustments to the treatment plan as needed.

## Discussion

India is often referred to as the "diabetes capital of the world" due to the high prevalence of diabetes in the population. Diabetic foot ulcers are a common complication of diabetes mellitus and can lead to significant morbidity and mortality if not properly managed. Given the significant burden of diabetic foot ulcers in India, there is a growing recognition of the importance of preventive measures such as regular foot examinations, patient education on foot care, and early intervention to prevent complications.

The use of ICG dye came out to be a revolutionary procedure in determining the prognosis of diabetic foot ulcers. ICGA can provide real-time visualization of blood flow in the microvasculature of the foot. By assessing perfusion using ICG, healthcare providers can identify areas of poor blood flow and tailor treatment strategies accordingly. This information can help in decision-making regarding revascularization procedures or wound management techniques. Overall, the use of ICG in diabetic foot ulcer management offers a noninvasive and dynamic approach to assess perfusion, evaluate tissue viability, monitor wound healing, and guide surgical interventions. Integrating ICG imaging into clinical practice can enhance the precision and effectiveness of treatment strategies for diabetic foot ulcers, ultimately improving outcomes for patients [10]. 

By reducing hypoxia and promoting angiogenesis, the study by Hajhosseini et al. shows that hyperbaric oxygen therapy significantly improves perfusion in chronic wounds. It also suggests that ICGA may play a part in the early detection of hyperbaric oxygen therapy recipients [11].

Another method for neutralizing microbes, particularly resistant bacterial biofilms, has been proposed: antimicrobial photodynamic therapy (A-PDT) or photodynamic antimicrobial chemotherapy (PACT). This study looked at the synergistic effects of PACT against common pathogens of diabetic foot ulcer infection, such as *Staphylococcus aureus* and *Pseudomonas aeruginosa*, in vitro. The fluorophores ICG and ethylenediamine tetraacetate (EDTA) combined with antibiotics mediated this effect [12].

ICG dye is an increasingly useful technique for measuring regional perfusion in diabetic feet is ICG dye. ICG dye parameters offer both quantitative and qualitative real-time visual views of perfusion in the area of interest. Compared to other techniques, ICGA is able to provide more information on regional perfusion [13].

The use of ICG-NIR in diabetic foot ulcers is a very novel technique which needs much more recognition in the medical fraternity. We selected this patient as he was very compliant with the procedure and had no issues being recorded for this case report.

The ICG dye being in infancy stage has its pros and cons which are mentioned here. The pros being that ICG emits NIR fluorescence when excited by specific wavelengths of light, allowing for real-time visualization of structures. This property is particularly useful in imaging techniques such as fluorescence angiography and lymphography. It has low risk of toxicity [13]. It is nonradioactive. It can be administered intravenously for vascular imaging, topically for ophthalmic procedures, or injected directly into tissues for intraoperative fluorescence guidance. The cons being that it is invasive and has limited tissue penetration. It has a high cost. It can bind to other proteins in the bloodstream giving background fluorescence. Further research is required for advocating the use of ICG in routine surgical practices.

## Conclusions

In this case report, we discussed about a noncompliant patient suffering from a diabetic foot ulcer who came out to be a classic case for a diagnostic approach using the ICG dye NIR technique. The ICG dye helped in differentiating the normally perfused and suboptimally perfused zone and eventually amputating the gangrenous part. ICG fluorescence angiography can be a valuable tool for assessing tissue perfusion in diabetic foot ulcers. It provides real-time information about blood flow, which can aid in decision-making regarding wound management and surgical interventions. While current evidence supports the use of ICG dye in diabetic foot ulcer management, ongoing research and risk-benefit analysis are necessary to optimize its utility, refine imaging techniques, and establish standardized protocols for its integration into clinical practice.
